# Associations between screen time, physical activity, and depressive symptoms during the 2019 coronavirus disease (COVID-19) outbreak among Chinese college students

**DOI:** 10.1186/s12199-021-01025-0

**Published:** 2021-11-02

**Authors:** Yi Zhang, Xiaoyan Wu, Shuman Tao, Shiyue Li, Le Ma, Yizhen Yu, Guilong Sun, Tingting Li, Fangbiao Tao

**Affiliations:** 1 Department of Maternal, Child and Adolescent Health, School of Public Health, Anhui Medical University, No. 81 Meishan Road, Hefei, 230032 Anhui China; 2 MOE Key Laboratory of Population Health Across Life Cycle, No. 81 Meishan Road, Hefei, 230032 Anhui China; 3 NHC Key Laboratory of Study on Abnormal Gametes and Reproductive Tract, No. 81 Meishan Road, Hefei, 230032 Anhui China; 4 Department of Nephrology, The Second Hospital of Anhui Medical University, 678 Furong Road, Hefei, 230601 Anhui China; 5 School of Health Sciences, Wuhan University, Wuhan, 430071 Hubei China; 6 School of Public Health, Xi’an Jiaotong University Health Science Center, 76 Yanta West Road, Xi’an, China; 7 Department of Maternal and Child Health, School of Public Health, Tongji Medical College, Huazhong University of Science & Technology, Hangkong Road 13, Wuhan, 430030 Hubei China; 8 South-Central Minzu University, 182 Minyuan Road, Wuhan, 430074 Hubei China

**Keywords:** Screen time, COVID-19ST, Physical activity, Depression, College students, COVID-19

## Abstract

**Background:**

The 2019 novel coronavirus disease (COVID-19) emerges in China, which spreads rapidly and becomes a public health emergency of international concern. Chinese government has promptly taken quarantine measures to block the transmission of the COVID-19, which may cause deleterious consequences on everyone’s behaviors and psychological health. Few studies have examined the associations between behavioral and mental health in different endemic areas. This study aimed to describe screen time (ST), physical activity (PA), and depressive symptoms, as well as their associations among Chinese college students according to different epidemic areas.

**Methods:**

The study design is cross-sectional using online survey, from 4 to 12 February 2020, 14,789 college students accomplished this online study, participants who did not complete the questionnaire were excluded, and finally this study included 11,787 college students from China.

**Results:**

The average age of participants was 20.51 ± 1.88 years. 57.1% of the college students were male. In total, 25.9% of college students reported depression symptoms. ST > 4 h/day was positively correlated with depressive symptoms (β = 0.48, 95%CI 0.37–0.59). COVID-19ST > 1 h/day was positively correlated with depressive symptoms (β = 0.54, 95%CI 0.43–0.65), compared with COVID-19ST ≤ 0.5 h/day. Compared with PA ≥ 3 day/week, PA < 3 day/week was positively associated with depression symptoms (β = 0.01, 95%CI 0.008–0.012). Compared with low ST and high PA, there was an interaction association between high ST and low PA on depression (β = 0.31, 95%CI 0.26–0.36). Compared with low COVID-19ST and high PA, there was an interaction association between high COVID-19ST and low PA on depression (β = 0.37, 95%CI 0.32–0.43). There were also current residence areas differences.

**Conclusions:**

Our findings identified that high ST or low PA was positively associated with depressive symptoms independently, and there was also an interactive effect between ST and PA on depressive symptoms.

## Background

In December 2019, COVID-19 confirmed cases first appeared in Wuhan, Hubei Province and quickly spread to many cities in China, has eventually become a public health emergency of international concern [1, 2]. The Chinese government has implemented strict self- and forced-quarantine measure across the country to hinder the transmission of the COVID-19 [3]. During the long-time home confinement, people’s life behaviors are likely to be changed largely, people’s psychological health may also be greatly affected [4].

As the previous studies testified, long-term home confinement leads to daily activities changed, which including decreased physical activity (PA) and increased screen time (ST), aims to seek accurate and up-to-date COVID-19 information in order to make informed decisions [5, 6]. In Xiang’s study, children and adolescents’ median time spent in PA decreased drastically, approximately 435 min/week decreased on average; frequency of physical inactivity extensively increased from 21.3 to 65.6% during COVID-19 epidemic [6]. Meanwhile, another recent study has reported that time spent in PA decreased by 2.30 ± 4.60 h/week and screen time increased by 4.85 ± 2.40 h/day among adolescents during COVID-19 epidemic [7]. In summary, these data demonstrated PA levels have decreased and screen time has increased since the COVID-19 outbreak among children and adolescents [6, 7]. The understanding of the effects of physical activity on mental health, therefore, has the potential to influence, in various aspects, the clinical practice of a psychologist or psychiatrist, on the one hand, as an auxiliary tool in the prevention and treatment of psychiatric diseases, and as a tool in the promotion of a more satisfactory quality of life, or on the other hand, as a cause of problems that require adequate diagnosis and effective treatment [8]. Previous research on screen time and mental health during COVID-19 has shown that excessive screen time is associated cardiovascular disease risk factors such as obesity, high blood pressure, and insulin resistance in youth [9] and high ST can cause various maladaptive psychophysiological responses, such as arousal of the central nervous system, and can also adversely association sleep patterns and intrapersonal social interactions [10, 11].

As COVID-19 outbreak is sudden and widespread, the overload of epidemic information (disinformation and false reports) through media may have a huge impact on disaster and emergency communications, which can make people feel panic, anxiety, worry, and depressed [12, 13]. Furthermore, changes in life behavior may disrupt normal rhythm of life and then consequently increase their mental health problems [14, 15], which will have a profound impact on the public’s mood and behavior during COVID-19 epidemic. In some longitudinal studies, adolescents maintaining regular physical activity (PA) showed an inverse association with psychosocial difficulties [16, 17]. Meanwhile, emerging persuasive evidence have shown that beneficial mental health of sufficient PA and adverse mental effects of screen time among children and youth [18, 19]. Compared to those who meet none or one movement behavior, children, and youth who meet all recommendations have better mental health [20].

Life behaviors have long been shown to play a role in the cause of chronic diseases, leading to suggestions for screen time, physical activity, and other behaviors among school-aged children and adults [21, 22]. However, there are few studies that investigate behavior changes among young adults during the COVID-19, as well as their effects on mental health among college students in different epidemic areas. Therefore, the present study first aims to describe the prevalence of ST, PA, and depressive symptoms among college students. Specifically, we also aim to evaluate the ST, PA, and depression symptoms in different epidemic areas. For these reasons, considering epidemic may well extend from weeks to months, drawing up an adapted physical training program and limited sedentary behaviors at home during the period of the epidemic, will decrease the negative psychological impact.

## Methods

### Setting and participants

**Fig. 1:**
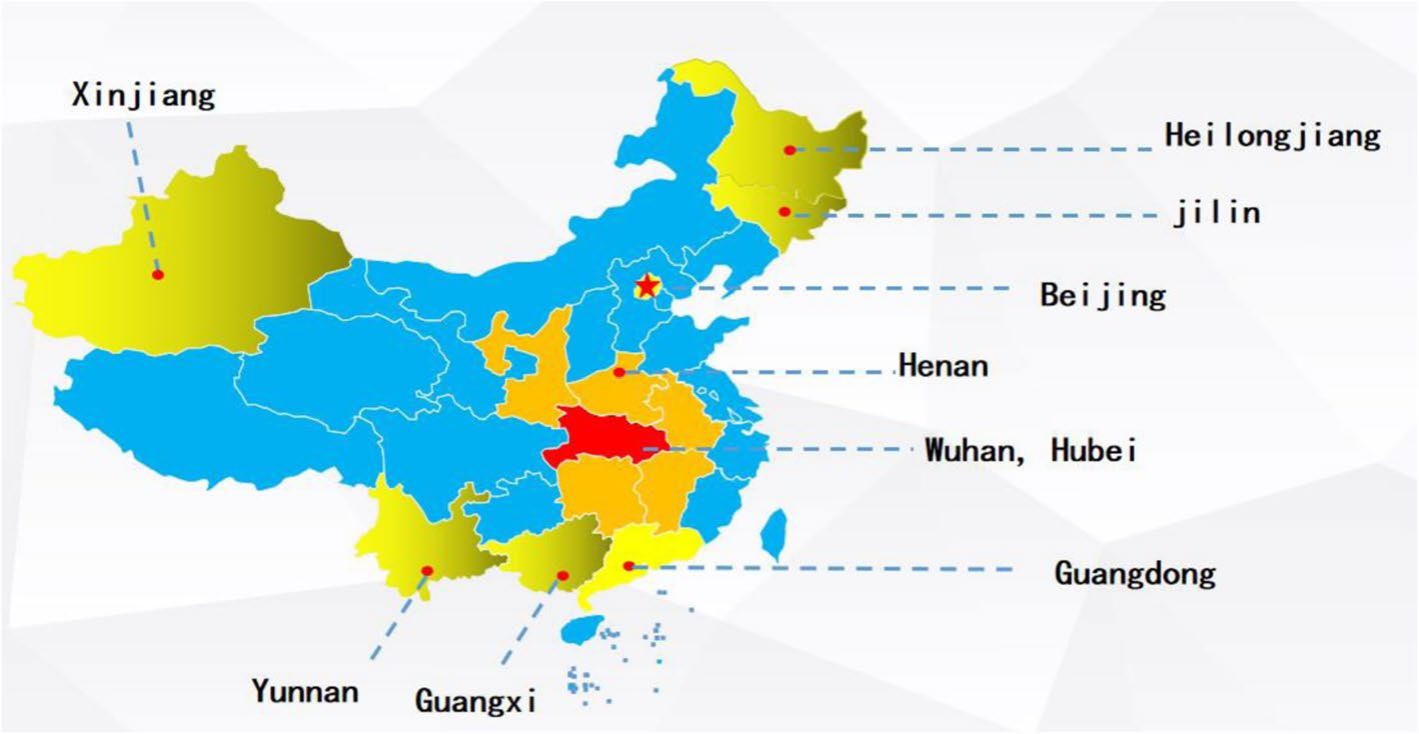
The distribution of sample with the locations of the 16 provinces

This nationwide cross-sectional online survey was conducted during 4 to 12 February 2020. A two-stage sampling strategy was used: at the first stage, according to the geographic location and cooperation intention, 16 provinces or municipalities were selected: Wuhan City, neighboring provinces of Hubei (Henan, Anhui, Jiangxi, Hunan, Chongqing, and Shanxi), first-tier cities (Beijing and Shanghai), and other provinces (Jiangsu, Guangdong, Guangxi, Yunnan, Xingjiang, Heilongjiang and Jilin) (see Fig. 1). A total of 4 universities were randomly selected in Wuhan, Hubei, and 15 universities were randomly selected from other provinces or municipalities. At the second stage, 100–120 students from each grade (in general, 5 years for medical students and 4 years for non-medical students) of a faculty were invited to participant online survey though Wenjuanxing platform (https://www.wjx.cn/). In total, 14,789 students were selected; finally, after excluding the participants without completed questionnaires, 11,787 participants from 16 cities, 19 colleges in China were involved in the current study.

### Procedure

They completed the questionnaires in Chinese college students through an online survey platform (Wenjuanxing platform). The Ethics Committee of Anhui Medical University approved this study (The number of the ethical approval is 20200319.). All respondents provided informed consent.

### Measurements

The study included some questions related to the COVID-19 outbreak. The structured questionnaire consisted of questions that covered several aspects: (1) sociodemographic data (gender, age, grade, types of faculty, current residence area); (2) screen time (ST) and physical activity (PA), time spent on COVID-19 news on social media (COVID-19 ST); (3) depressive symptoms. ST was measured with one question: “How many hours per day did you spend on the computer (including playing video games or computer games or using a computer for something else) and watching TV/video programs during the past 7 days?” [20]. ST was categorized as > 4 h/day (high), 2–4 h/day (medium) and ≤ 2 h/day (low) [23–25]. COVID-19ST was measured with one question: “How much time do you spend reading or watching COVID-19 on your phone, computer, or TV every day (COVID-19 ST) during the past 7 days?”. The PA of participants was measured with one question, “How many days have you been physical activity for at least one hour (60 min) in the last week? (that is, the total amount of physical activity in a day that makes your heart beat faster and sometimes makes your breathing significantly faster)?” [26]. High PA was defined as at least 3 days per week (PA ≥ 3 days) of exercise [27]. This item has been previously used in recognized student surveys such as the WHO’s Health Behaviour in School-aged Children (HBSC) survey, and has been shown to produce valid and reliable responses [28].

Depressive symptoms were measured using the patient health questionnaire (PHQ) and calculations of scores were based on the previous study [29, 30]. The total stress subscale score was divided into normal (0–4), mild stress (5–9), moderate stress (10–14), severe stress (15–21) [31]. The questionnaire used in our study has previously been well validated [32].

### Statistical analysis

Descriptive statistics of sociodemographic characteristics, screen time, physical activity were are presented as frequencies (*n*) and percentages (%). Chi-square test was used to analyze the detection rates of mild and moderate depression in general data, ST, COVID-19ST, and PA. The scores of the PHQ-9 scale were expressed as a dichotomous variable. To examine the interaction between PA, ST, and COVID-19ST on depression, we created a variable for the various combinations of PA and ST categories: high PA + low ST, high PA + high ST, and high PA + medium ST, low PA + low ST, low PA + high ST, and low PA + medium ST; and high PA + low COVID-19ST, high PA + high COVID-19ST, and high PA + medium COVID-19ST, low PA + low COVID-19ST, low PA + high COVID-19ST, and low PA + medium COVID-19ST. We used linear regressions to examine the association between ST, COVID-19ST, physical activity, and the depression symptoms, with a significance level of *P* < 0.05. The associations are presented in odds ratios and their 95% confidence intervals (CIs). Statistical analysis was performed using SPSS Statistic 23.0.

## Results

### Characteristics of respondents

Our study included 11,787 college students from 16 cities, 19 colleges in China. The demographic characteristics of college students were shown in Table 1. Their mean age was 20.51 ± 1.88 years. 57.1% of the students were female. 25.9% (3053/11,787) students reported depressive symptoms, 31.3% (187/597), 26.0% (1,297/4,987), 22.7% (243/1,070), and 25.8% (1,326/5,133) in Wuhan area (Area 1), other cities in Hubei (Area 2), neighboring provinces of Hubei (Area 3), and other areas (Area 4), respectively. Area 1 was statistically higher than other three areas (*χ*^2^ = 25.57). The percentage of COVID-19ST > 1 h/day, 0.5–1 h/day, and ≤ 0.5 h/day among college students were 33.4%, 39.2%, 27.4%, and the PA < 3 day/week and PA ≥ 3 day/week accounted for 70.7% and 29.3%. Higher rate of depressive symptoms was found in students who reported higher screen time (≥ 4 h vs 2–4 h mild: 20.2% vs 15.1%, moderate: 6.3% vs 4.2%, moderate-severe: 3.5% vs 1.5%, severe: 1.3% vs 0.5% ; *χ*^2^ = 196.15, *P* < 0.001); higher rate of depressive symptoms was found in students who reported higher screen time (≥1 h vs 0.5–1 h mild: 19.3% vs 17.4%, moderate: 6.0% vs 4.3%, moderate–severe: 4.1% vs 2.1%, severe: 1.6% vs 0.6% ; *χ*^2^ = 119.90, *P* < 0.01); or lower physical activity (mild 18.4% vs 14.2%, moderate 5.6% vs 2.9%, moderate-severe 3.1% vs 2.3%, severe 1.1% vs 0.9%, *χ*^2^ = 95.27, *P* < 0.001). The rates of depressive symptoms were 31.3%, 20.3%, 22.0% in ST > 4 h/day group, 2–4 h/day group, and ≤ 2 h/day group. The rates of depressive symptoms were 31.0%, 24.4%, and 21.8% in COVID-19ST > 1 h/day group, 0.5–1 h/day group and ≤ 0.5 h/day group. Depressive symptoms accounts for 28.2% and 20.1% in PA < 3 day/week group and PA ≥ 3 day/week group. These results were all showed in Table 1.

**Table 1:** Depression prevalence of college students with different demographic characteristics, *n*(%)

Demographic variables	Total	Depression					*χ*^2^
		Prevalence	Mild	Moderate	Moderate-severe	Severe	
Gender							
Male	5056(42.9)	1131(22.4)	709(14.0)	208(4.1)	171(3.4)	43(0.9)	84.35^**^
Female	6731(57.1)	1922(28.5)	1313(19.5)	361(5.4)	171(2.5)	77(1.1)	
Residential areas							
Rural	5660(48.0)	1431(25.3)	942(16.6)	286(5.1)	153(2.7)	50(0.9)	6.79
Urban	6127(52.0)	1622(26.4)	1080(17.6)	283(4.6)	189(3.1)	70(1.1)	
Age (years)							
≤ 19	3860(32.7)	950(24.6)	667(17.3)	165(4.3)	85(2.2)	33(0.9)	27.47^**^
20–22	6426(54.5)	1746(27.2)	1116(17.4)	333(5.2)	224(3.5)	73(1.1)	
≥ 23	1501(12.7)	357(23.8)	239(15.9)	71(4.7)	33(2.2)	14(0.9)	
Current residence areas							
Area 1	597(5.1)	187(31.4)	114(19.1)	38(6.4)	23(3.9)	12(2.0)	25.57^*^
Area 2	2237(19.0)	579(25.9)	409(18.3)	97(4.3)	61(2.7)	12(0.5)	
Area 3	2750(23.3)	718(26.1)	481(17.5)	134(4.9)	75(2.7)	28(1.0)	
Area 4	6203(52.6)	1569(25.3)	1018(16.4)	300(4.8)	183(3.0)	68(1.1)	
Type of faculty							
Medical students	5770(49.0)	1338(19.2)	898(15.6)	255(4.4)	134(2.3)	51(0.9)	46.87^**^
Non-medical students	6017(51.0)	1715(28.5)	1124(18.7)	314(5.2)	208(3.5)	69(1.1)	
Grade							
Freshmen	2930(24.9)	708(24.2)	484(16.5)	134(4.6)	67(2.3)	23(0.8)	22.35
Sophomore	2609(22.1)	707(27.2)	453(17.4)	137(5.3)	85(3.3)	32(1.2)	
Junior	2667(22.6)	724(27.1)	481(18.0)	123(4.6)	91(3.4)	29(1.1)	
Senior	2314(19.6)	619(26.8)	407(17.6)	117(5.1)	71(3.1)	24(1.0)	
Fifth-grade	1267(10.7)	295(23.2)	197(15.5)	58(4.6)	28(2.2)	12(0.9)	
ST							
> 4 h	5570(47.3)	1741(31.3)	1126(20.2)	349(6.3)	195(3.5)	71(1.3)	196.15^**^
2–4 h	3706(31.4)	756(20.4)	560(15.1)	119(3.2)	57(1.5)	20(0.5)	
≤ 2 h	2511(21.3)	556(22.1)	336(13.4)	101(4.0)	90(3.6)	29(1.2)	
COVID-19ST							
> 1 h	3933(33.4)	1220(31.0)	758(19.3)	237(6.0)	161(4.1)	64(1.6)	119.90^**^
0.5–1 h	4623(39.2)	1129(24.4)	803(17.4)	201(4.3)	96(2.1)	29(0.6)	
≤ 0.5 h	3231(27.4)	704(21.8)	461(14.3)	131(4.1)	85(2.6)	27(0.8)	
PA							
≥ 3 day	3453(29.3)	695(20.1)	491(14.2)	99(2.9)	80(2.3)	25(0.7)	95.27^**^
< 3 day	8334(70.7)	2358(28.3)	1531(18.4)	470(5.6)	262(3.1)	95(1.1)	

Wuhan (Area 1), other cities in Hubei (Area 2), neighboring provinces of Hubei (Area 3), and other provinces (Area 4); *PA* physical activity, *ST* screen time

^*^P < 0.05

^**^P < 0.01

There were some differences between ST, PA, and demographic characteristics. Among gender, female students had higher ST who reported > 4 h/day (*χ*^2^ = 225.13, *P* < 0.01), and had PA < 3 day/week (*χ*^2^ = 80.08, *P* < 0.01), had COVID-19ST > 1 h/day (*χ*^2^ = 110.80, *P* < 0.01). There were others demographic characteristics on ST and PA. However, there was no difference between current residence areas and ST, PA, but there was association between current residence areas and COVID-19ST (*χ*^2^ = 20.86, *P* < 0.01). Other results were shown in Table 2.

**Table 2:** The prevalence of screen time, time spent on COVID-19 news on social media and physical activity with different demographic characteristics

Variables	Total	ST, *n* (%)				PA, n (%)			COVID-19ST, *n* (%)			
		> 4 h	2–4 h	≤ 2 h	*Chi-square value*	< 3 days	≥ 3 day	*Chi-square value*	> 1 h	0.5–1 h	≤ 0.5 h	*Chi-square value*
Gender												
Male	5056	2,104(41.6)	1,557(30.8)	1,395(27.6)	225.13^**^	3356(66.4)	1700(33.6)	80.08^**^	1478(29.2)	1965(38.9)	1613(31.9)	110.80^**^
Female	6731	3466(51.5)	2149(31..9)	1116(16.6)		4978(74.0)	1753(26.0)		2455(36.5)	2658(39.5)	1618(24.0)	
Residential areas												
Rural	5660	2,541(44.9)	1776(31.4)	1343(23.7)	42.92^**^	1707(30.2)	3953(69.8)	3.92^*^	1727(30.5)	2322(41.0)	1611(28.5)	40.02^**^
Urban	6127	3029(49.4)	1930(31.5)	1168(19.1)		1746(28.5)	4381(71.5)		2206(36.0)	2301(37.6)	1620(26.4)	
Age (years)												
≤ 19	3860	1816(47.0)	1262(32.7)	782(20.3)	13.38^*^	2664(69.0)	1196(31.0)	15.03^**^	1045(27.1)	1549(40.1)	1266(32.8)	140.39^**^
20–22	6426	3,090(48.1)	1960(30.5)	1376(21.4)		4554(70.9)	1872(29.1)		2259(35.6)	2516(39.2)	1621(25.2)	
≥ 23	1501	664(44.2)	484(32.2)	353(23.5)		1116(74.4)	385(25.6)		599(39.9)	558(37.2)	344(22.9)	
Current residence areas												
Area 1	597	301(50.4)	183(30.7)	113(18.9)	11.54	423(70.9)	174(29.1)	6.92	239(40.0)	191(32.0)	167(28.0)	20.86^**^
Area 2	2237	1101(49.2)	692(30.9)	444(19.8)		1555(69.5)	682(30.5)		710(31.7)	903(40.4)	624(27.9)	
Area 3	2750	1312(47.7)	847(30.8)	591(21.5)		1997(72.6)	753(27.4)		888(32.3)	1096(39.9)	766(27.9)	
Area 4	6203	2856(46.0)	1984(32.0)	1363(22.0)		4359(70.3)	1,844(29.7)		2096(33.8)	2433(39.2)	1674(27.0)	
Students type												
Medical students	5770	2808(48.7)	1807(31.3)	1155(20.0)	13.58^**^	4205(72.9)	1565(27.1)	25.74^**^	1994(34.6)	2316(40.1)	1460(25.3)	25.56^**^
Non-medical students	6017	2762(45.9)	1899(31.6)	1356(22.5)		4129(68.6)	1888(31.4)		1939(32.2)	2307(38.3)	1771(29.4)	
Grade												
Freshmen	2930	1276(43.5)	1017(34.7)	637(21.7)	31.30^**^	1973(67.3)	957(32.7)	59.57^**^	726(24.8)	1180(40.3)	1024(34.9)	246.14^**^
Sophomore	2609	1263(48.4)	797(30.5)	549(21.0)		1833(70.3)	776(29.7)		817(31.3)	1050(40.2)	742(28.4)	
Junior	2667	1272(47.7)	799(30.0)	596(22.3)		1847(69.3)	820(30.7)		932(34.9)	1053(39.5)	682(25.6)	
Senior	2314	1114(48.1)	716(30.9)	484(20.9)		1690(73.0)	624(27.0)		907(39.2)	872(37.7)	535(23.1)	
Fifth-grade	1267	645(50.9)	377(29.8)	245(19.3)		991(78.2)	276(21.8)		551(43.5)	468(36.9)	248(19.6)	

Wuhan (Area 1), other cities in Hubei (Area 2), neighboring provinces of Hubei (Area 3), and other provinces (Area 4); *PA* physical activity, *ST* screen time

^*^P < 0.05

^**^P < 0.01

### The correlation of screen time and physical activity with depression symptoms during COVID-19 outbreak

In Table 3, after adjusting for confounding factors, compared with daily ST ≤ 2 h/day, ST > 4 h/day was positively correlated with depressive symptoms (*β* = 0.48, 95%CI 0.37–0.59). After adjusting for confounding factors, compared with daily COVID-19ST ≤ 0.5 h/day, COVID-19ST > 1 h/day was positively correlated with depressive symptoms (*β* = 0.54, 95%CI 0.43–0.65). After adjusting for confounding factors, compared with PA < 3 day/week, weekly PA ≥ 3 day/week was positively correlated with depressive symptoms (*β* = 0.01, 95%CI 0.008–0.012). After adjusting for confounding factors, compared with low ST and high PA, higher ST and low PA were more likely to cause depressive symptoms (*β* = 0.31, 95%CI 0.26–0.36). After adjusting for confounding factors, compared with low COVID-19ST and high PA, higher COVID-19ST and low PA were more likely to cause depressive symptoms (*β* = 0.37, 95%CI 0.32–0.43).

**Table 3:** The individual and interactive between screen time, COVID-19 screen time, physical activity, and depression among college students

Different behavior variables	Depression		
	*R*^*2*^	*F*	*Β*(95%)
ST	0.017	25.24	0.48(0.37,0.59)^**^
COVID-19ST	0.018	27.43	0.54(0.43,0.65)^**^
PA	0.018	27.05	0.01(0.008,0.012)^*^
ST*PA	0.022	33.69	0.31(0.26,0.36)^**^
COVID-19ST*PA	0.025	38.34	0.37(0.32,0.43)^**^

*PA* physical activity, *ST* screen time

^*^P < 0.05

^**^P < 0.01

### The relationship between screen time, physical activity, and depression symptoms among college students in different epidemic areas

Table 4 showed the correlation between ST, PA, and depression symptoms in four different areas among college students. After adjusting for confounding factors, in different areas, ST and PA were significantly correlated with depression symptoms. Area 1: high ST (*β* = 1.265, 95%CI 0.72–1.81), low PA (*β* = 0.014, 95%CI 0.004–0.025). Area 2: high ST (*β* = 0.69, 95%CI 0.47–0.92); low PA (*β* = 0.009, 95%CI 0.005–0.013); Area 3: high ST (*β* = 0.20, 95%CI 0.15–0.42); low PA (*β* = 0.008, 95%CI 0.003–0.012); Area 4: high ST (*β* = 0.46, 95%CI 0.31–0.61); low PA (*β* = 0.011, 95%CI 0.008–0.014). COVID19-ST was also had same results. They also had a multiplied interaction impact (ST and PA, COVID-19ST and PA) on depression symptoms. There were shown in Table 4.

**Table 4:** The individual and interactive between screen time, COVID-19 screen time, physical activity, and depression among college students in different epidemic areas

Different behavior variables	Depression		
	*R*^*2*^	*F*	*Β*(95%)
Area1			
ST	0.068	6.15	1.265(0.72,1.81)^**^
COVID-19ST	0.648	4.197	0.51(0.28,0.74)^**^
PA	0.048	4.198	0.014(0.004,0.025)^*^
ST*PA	0.065	5.82	0.54(0.29,0.79)^**^
COVID-19ST*PA	0.06	5.38	0.55(0.28,0.83)^**^
Area2			
ST	0.02	6.48	0.69(0.47,0.92)^**^
COVID-19ST	0.012	4.002	0.51(0.28,0.74)^**^
PA	0.012	8.743	0.009(0.005,0.013)^*^
ST*PA	0.021	6.983	0.33(0.23,0.44)^**^
COVID-19ST*PA	0.02	6.46	0.34(0.23,0.46)^**^
Area3			
ST	0.019	7.51	0.20(0.15,0.42)^**^
COVID-19ST	0.029	11.569	0.64(0.42,0.87)^**^
PA	0.022	8.743	0.008(0.003,0.012)^*^
ST*PA	0.023	9.042	0.20(0.096,0.31)^*^
COVID-19ST*PA	0.025	22.58	0.35(0.30,0.45)^**^
Area4			
ST	0.016	14.16	0.46(0.31,0.61)^**^
COVID-19ST	0.016	14.32	0.48(0.33,0.63)^**^
PA	0.019	16.942	0.011(0.008,0.014)^*^
ST*PA	0.023	20.536	0.32(0.25,0.39)^**^
COVID-19ST*PA	0.025	22.58	0.37(0.30,0.45)^**^

*PA* physical activity, *ST* screen time

^*^P < 0.05

^**^P < 0.01

## Discussion

Our nationwide survey had some main findings. Firstly, nearly 50.0% of the college students ST were more than 4 h per day, 33.4% of the college students spent time on COVID-19ST, and 70.7% of the college students had inadequate PA (PA < 3 day/week). Secondly, in our study, 25.9% of college students reported the depression symptoms. Among them, 31.3%, 26.0%, 22.7%, and 25.8% of depression symptoms were found in Wuhan (Area 1), other cities in Hubei (Area 2), neighboring provinces of Hubei (Area 3), and other provinces (Area 4), respectively. Finally and interestingly, we also found the independent and interactive association between PA, ST, and depression symptoms in different epidemic area. To the best of our knowledge, this is the first large population-based study examining media (screen time) and physical activity-related psychological impact during the epidemic outbreak among college students.

During COVID-19 epidemic, the present study find young adults spending ≥ 4 h per day in ST was 47.3%, especially in Wuhan area (50.4%), also during the epidemic, some researchers have demonstrated that ST was higher in adults, the mean ST was 264 min/day [5]; and one-third of community-based adults and health professionals spending ≥ 2 h per day in ST [33]. It also showed the extent to which the epidemic had hit people, and the results of college students were higher than previous study [27]. Most previous studies examined the ST exposure for other age-group, while our study focused on college students, who were more prone to susceptibility about “infodemic” [34]. Meantime, in our study, we find youths insufficient PA was nearly 71%, which was higher than adults (nearly 60%) during the epidemic [5], while the prevalence of insufficient physical activity was higher than previous study level (51%) among college students [35].

The COVID-19 epidemic had caused serious threats to people’s physical health and lives [36]. It has also triggered a wide variety of psychological problems, such as panic disorder, anxiety and depression [12, 13]. The depression symptoms of this study were higher than the general survey of college students [37, 38] and another cross-sectional epidemiological study [39]. Another study had reported that in a longer duration of quarantine, a high prevalence of invisible psychological pressure caused by screen time was positively correlated with depressive symptoms [40]. There results all demonstrated that the psychological impact of the epidemic cannot be ignored, because in previous study, youths showed significantly higher rates of posttraumatic stress disorder (PTSD) symptoms because they have experienced quarantine [41]. The same results have been replicated in animal experiments. Even a brief duration (e.g., 24 h) of isolation in adolescent rodents can cause increased anxiety [42], hyperactivity [43], and heightened sensitivity to social rewards [44], which extends to the seeking of food or drug rewards, making these animals particularly prone to developing addictions [45]. Specially, when rodent adolescent isolation occurs chronically, over 1 week or longer, it has even more profound effects [46]. Moreover, more attention should be paid to behaviors and the depression symptoms of females, non-medical background, and college students in Wuhan area. Females were more prone to high ST, low PA, and depression symptoms than males perhaps because women are more perceptual, emotional, relatively vulnerable to tension, and has high incidence of depression symptoms than men, as reported in the literature [5, 12, 47, 48].

The findings of this study suggested that high ST and low PA were significant positively correlated with depression symptoms individually and interactively. Some studies have also found high ST could cause positive relationships with anxiety and depression, so sedentary screen time among young adults are also significant factors to assess health behaviors [49, 50]. During COVID-19, what we acquired information through social media (screen time) is sharply increased quantity but quality is uncontrolled; it is also accompanied by fragmented information [51, 52], and that is where the adverse emotion is also increased [51, 53, 54]. Fragmentation of information fragmentation of news will only lead to unnecessary trouble. Accumulating evidence suggest that screen time exposure of traumatizing or threatening content can influence fear conditioning by activating fear circuitry in the brain and can produce PTSD symptoms, especially flash backs [54]. Interestingly, we also found medium ST was negatively correlated with college students’ depression symptoms, one possible reason was that in COVID-19 outbreak, many people are concerned with the COVID-19-related news and information including confirmed patients’ information and their movements, precautionary measures about COVID-19 through media and Internet, which could be provided for online information, i.e., Ali Health, Ping An Good Doctor, Ding xiang yuan forum, WeChat implemented in China) and restored daily routines (i.e., online course, online working, exercise items on electronic devices) [33], and also suggested a U-shaped non-linear association between ST and depression symptoms [55]; appropriate information-seeking behaviors may reduce psychological health caused by uncertainty during COVID-19 epidemic [56], similarly, relative studies have also testified that to some extent, social media, for example engaging in directed communication (i.e., messaging), have been shown to increase well-being [57]; yet caution is warranted toward spending excessive time searching for COVID-19 news on screen time given the infodemic and emotional contagion through online social networks [33].

In additionally, increasing evidences were suggested that high PA was associated with numerous health benefits [58, 59], and also have positive effect during COVID-19 [60]. PA can influence the mental health of college students [61, 62]. Lower physical activity led to more depression, which was consistent with the previous studies [27]. There were similar results between PA and depression symptoms during COVID-19 epidemic [63, 64]. Associations between reduced depression symptoms, lower PA levels and increased ST in adolescents have been reported [65–68]. Previous studies concerned about people have higher depression symptoms who do not meet the recommendation ST ≤ 2 h/day and weekly PA > 3 days [66].

In our study, we also posed different ST and COVID-19ST divisions, according to the special situation in COVID-19, we found that the interaction between ST > 4 h/day, weekly PA < 3 days and COVID-19ST > 1 h/day, weekly PA < 3 days were also correlated with higher depression symptoms. So our study suggested that high ST and low PA increase the risk of depression symptoms independently and also synergistically in college students. Some studies have suggested possible mechanisms: metabolic psychological health status 18) or social withdrawal [69]. Another explanation was that ST displaced PA [5, 70], which has proven to be protective against depressive and anxiety disorders [71, 72]. Especially in the case of public health emergencies, with the continuous influx of information (whether negative or positive), what is more, the negative information will be magnified, further influence PA, so the ST and PA behaviors of college students will be affected to different degrees, therefore cause unpredictable outcomes [5]. Some possible reasons were that high ST may displace their study and PA time, and then further infect their physical and psychosocial health [73–75], in a similar way, PA could buffer consequences of high screen time [76] and then further promote positive association of psychological well-being. It also named “displacement hypothesis” [73, 77, 78]. So, we should reasonably organize our time. Other study also demonstrated that maintaining opportunities for outdoor exercise and limiting screen time may promote better mental and general health during periods of confinement. In addition, taking into account different endemic areas, we can increase prevention and control and technical guidance to those areas.

We also explored the differences between screen time and physical activity of college students in different epidemic areas. The results showed that compared with college students in the other three areas, the higher ST and the lower PA in Area 1 college students, have higher the depression symptoms. The reasons may include that Area 1 students, considering that they live locally, will pay close attention to the situation of the outbreak, so they will have more screen time, which will lead to more depression symptoms. Also, due to the reason for home quarantine, physical activity was naturally decreased in four areas, especially in Area 1 [5]. In combination with the increase of screen time, physical activity would further increase the occurrence of depression.

## Strengths and limitation

Our study also has some limitations. Firstly, the current study was a cross-sectional study, which could not build a causal relation between life behavior and psychological health outcomes. Furthermore, ST and PA were self-reported, considering special epidemic situations, we cannot actually test college students, so there may be a recall bias. Thirdly, the mechanism underlying the association was not directly assessed. Future longitudinal studies should further explore possible psychosocial and biological pathways. Finally, we had not obtain mental health conditions and life behaviors before the outbreak. Moreover, our study is a continuous follow-up study, which a follow-up investigation every 3 months will be conducted, thus enabling to further clarify the relationship.

Even though these limitations, our study also is also worthy to attention. Firstly, we have large samples and different epidemic areas to compare regional difference; therefore, local schools and governments can respond in time. Secondly, we timely grasp the epidemic problem, for domestic college students to provide some reference psychological research, especially the psychological factors. Thirdly, during the quarantine period, on the one hand, people should not only follow the news of the epidemic through the media, so as to further understand the current situation; finally, on the other hand, because of the quarantine, people's activities are greatly restricted, especially those who are in contact with the confirmed cases, and home quarantine will have different degrees of psychological impact. Therefore, our study took this as a starting point to conduct a nationwide survey on the mental health of college students.

## Conclusion

Our findings identified that high ST and low PA exposure were positively associated with depression symptoms, and there was also an interactive effect. Compared with the emerging Wuhan City, other epidemic regions have lower risk of psychological health problems. In a major public health emergency like this, the media plays an important role in the society by providing authoritative information and social support and helping isolated individuals feel connected [79]. Relevant government departments should focus on the non-medical background of health education of college students by improving their awareness of COVID-19 [80, 81]. Physical activity is an important public health tool used in the treatment and prevention of various physical diseases, as well as in the treatment of some psychiatric diseases such as depressive and anxiety disorders [82]. Meanwhile, relevant government departments and universities should promote positive influence [83] to attract college students to reasonably arrange screen time and physical activity during the epidemic. Thus, during the epidemic, a better understanding of the association of PA, ST, and among college students will help school leaders and the Ministry of Education urgently identify and implement effective policies and interventions for college students [6]. COVID-19 as a pandemic is beyond the scope of the biomedical approach. It is a phenomenon that requires a biopsychosocial exploration as the factors influencing its proliferation and increased burden are multifaceted. The significance of our research is to provide an idea for teenagers to arrange their time reasonably when facing major public health emergencies (such as COVID-19), so as to deal with psychological problems during isolation and to establish ways for schools and government agencies to deal with public health events and prevent mental health. The significance of our research is to provide an idea for teenagers to arrange their time reasonably when facing major public health emergencies (such as COVID-19), so as to deal with psychological problems during isolation and to establish ways for schools and government agencies to deal with public health events and prevent mental health. Lack of PA and excessive ST were relevant to adiposity among rural Chinese adolescents [84] and social skill, which was one component of psychosocial wellbeing [85]. In addition, taking into account different endemic areas, we can increase prevention and control and technical guidance to those areas. Implementing a screen time and physical activity policy or national plan, establishing a public health screen time ad physical activity team, integrating screen time, and physical activity monitoring into school demographic health surveys and supporting multisectoral collaboration is a feasible and most cost-effective strategy to help the country move towards a more active and less sedentary population and society.

## Data Availability

The data used and analyzed during the study are available from the corresponding author on reasonable request.

## Abbreviations

ST: Screen time
PA: Physical activity
COVID-19: 2019 coronavirus disease
PHQ: Patient Health Questionnaire
